# Supplementary material for: Iterative immunogen optimization to focus immune responses on a conserved, subdominant viral epitope
Source: bioRxiv. 2025 Nov 6:2025.11.04.686334. Preprint. [Version 1] doi: 10.1101/2025.11.04.686334 (PMC12637436; doi:10.1101/2025.11.04.686334)
Supplement: 1 — Supplemental Figure 1. Subdominant, conserved HA epitope targeted by broadly cross-reactive mAbs. (Left) Structure of the HA1 domain of the pre-fusion HA trimer (individual monomers: grey, pale orange, and pale blue; A/Hong Kong/1/1968, PDBid:4FNK37), with the 220-loop (red) highlighted and key epitope residues P221 and V223 (sticks), which are partially occluded on pre-fusion HA. (Right) Structure of HA head (pale orange, A/Hong Kong/1/1968) bound to FluA-20 mAb (green/blue) (PDBid:6OCB7) with key 200-loop epitope residues P221, V223, and R229 (sticks). (Lower) Computational model of HA220 (grey) with the grafted epitope shown in red, with key 220-loop epitope residues P221, V223, and R229 (sticks) completely exposed. Supplemental Figure 2. BLI binding curves of HA220 immunogens interacting with target mAbs. Supplemental Figure 3 Binding of HA220 scaffolds to mabs measured by ELISA. ELISA binding data of HA220 RS and MPNN immunogens to FluA-20. [file NIHPP2025.11.04.686334v1-supplement-1.pdf]

bioRxiv preprint doi: https://doi.org/10.1101/2025.11.04.686334; this version posted November 6, 2025. The copyright holder for this preprint (which was not certified by peer review) is the author/funder, who has granted bioRxiv a license to display the preprint in perpetuity. It is made available under aCC-BY 4.0 International license.

Supplemental Table 1

	HA220-7 + D2 H1-1/H3-1 H3 Fab	HA220-M4C + S1V2-58 Fab
Data collection		
Space group	$P4_122$	$C2_1$
Cell dimensions		
a, b, c (Å)	110.9, 110.9, 164.8	238.6, 158.9, 136.7
α, β, γ (°)	90.0, 90.0, 90.0	90.0, 124.9, 90.0
Resolution (Å)	82.43-2.40 (2.49-2.40)	71.11-3.90 (4.07-3.90)
R_{merge}	0.188 (1.931)	0.243 (0.957)
**I/σI**	9.7 (1.6)	4.2 (1.7)
CC1/2	0.997 (0.780)	0.931 (0.357)
Completeness (%)	100.0 (100.0)	99.7 (99.9)
Redundancy	12.8 (12.4)	3.3 (3.5)
Refinement		
R_{work}/R_{free} (%)	21.5/24.3	21.7/27.6
No. atoms		
Protein	4466	18082
Ligand	0	0
Water	130	0
Average B-factors		
Protein	61.3	112.0
Solvent	55.6	
R.m.s deviations		
Bond lengths (Å)	0.003	0.003
Bond angles (Å)	0.58	0.69
Clashscore	2.59	4.76
Ramachandran		
Favored (%)	97.7	95.1
Allowed (%)	2.3	4.6
Outliers (%)	0.0	0.3

Supplementary Figure 1

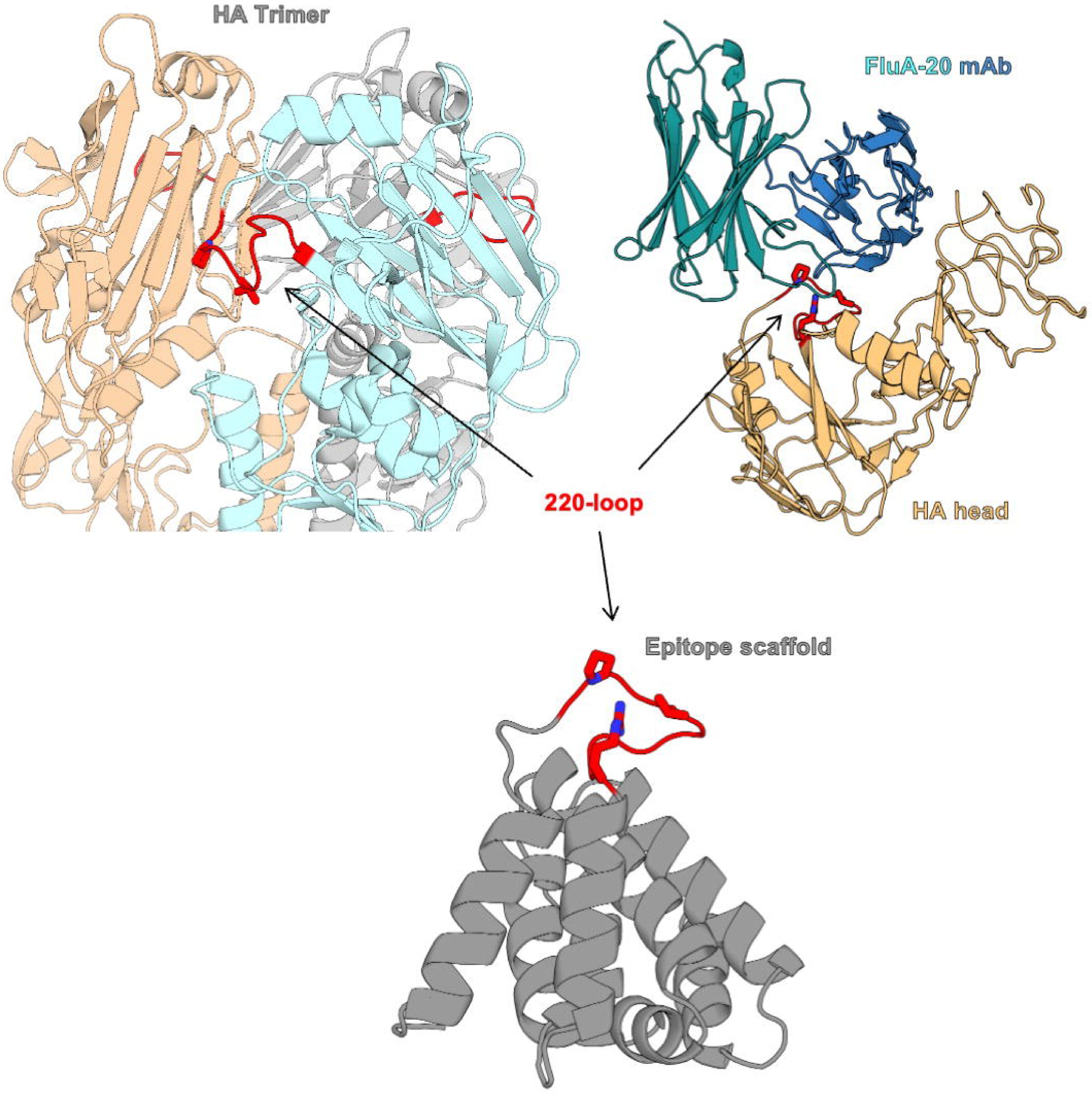

bioRxiv preprint doi: https://doi.org/10.1101/2025.11.04.686334; this version posted November 6, 2025. The copyright holder for this preprint (which was not certified by peer review) is the author/funder, who has granted bioRxiv a license to display the preprint in perpetuity. It is made available under aCC-BY 4.0 International license.

Supplementary Figure 2

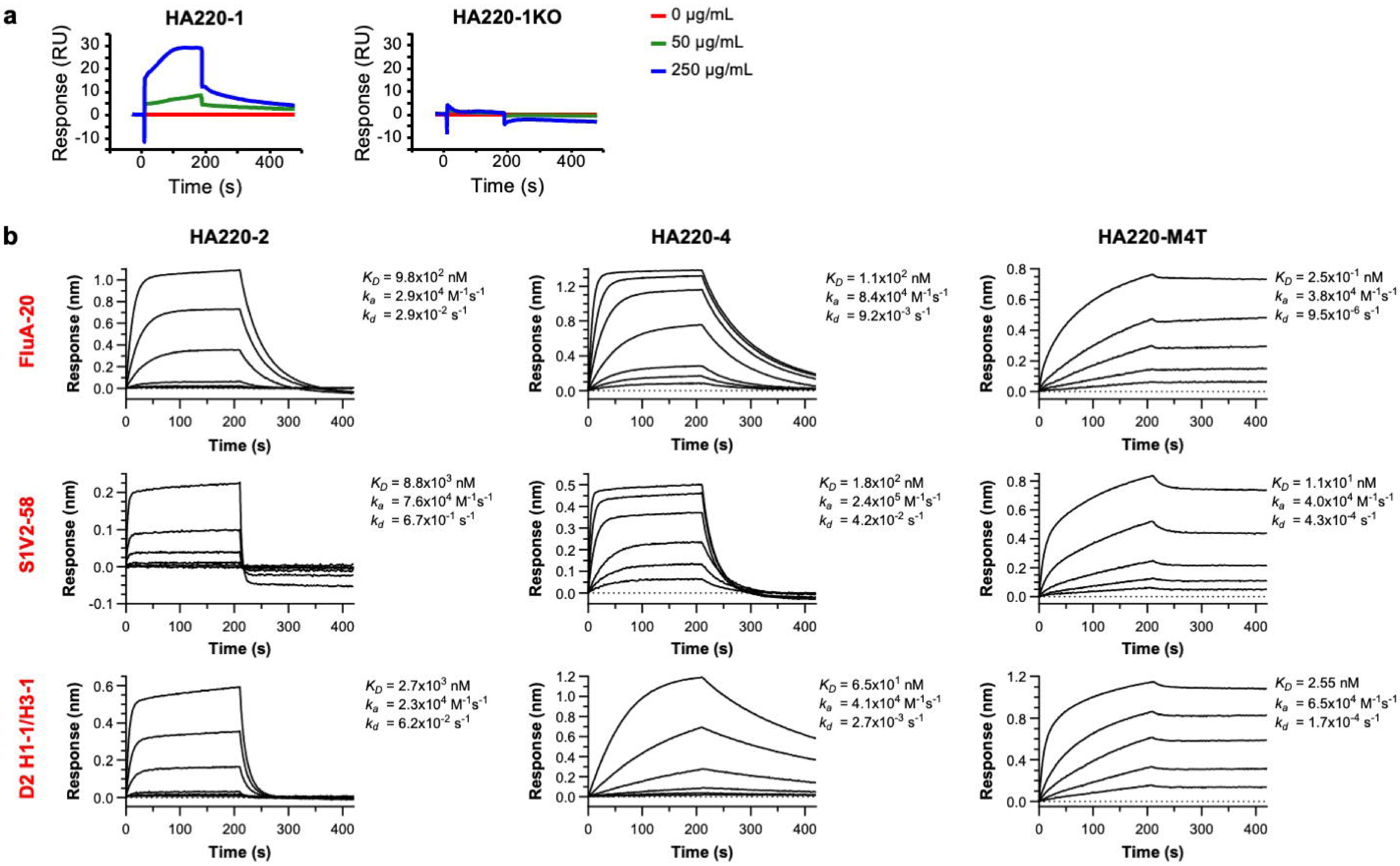

bioRxiv preprint doi: https://doi.org/10.1101/2025.11.04.686334; this version posted November 6, 2025. The copyright holder for this preprint (which was not certified by peer review) is the author/funder, who has granted bioRxiv a license to display the preprint in perpetuity. It is made available under aCC-BY 4.0 International license.

Supplementary Figure 3

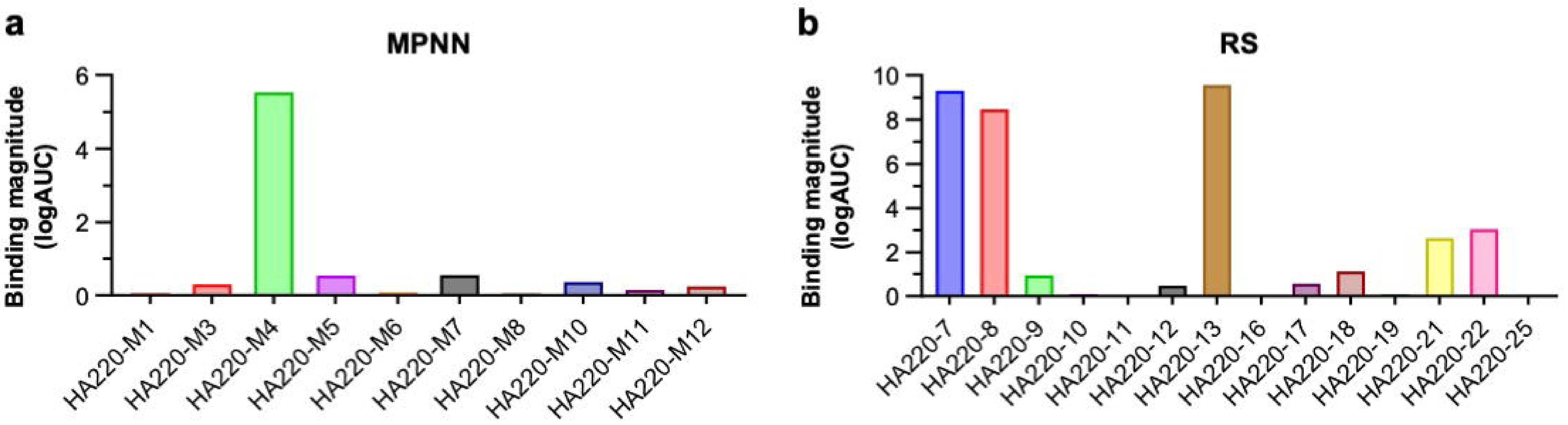

bioRxiv preprint doi: https://doi.org/10.1101/2025.11.04.686334; this version posted November 6, 2025. The copyright holder for this preprint (which was not certified by peer review) is the author/funder, who has granted bioRxiv a license to display the preprint in perpetuity. It is made available under aCC-BY 4.0 International license.
